# SimuVet: a preliminary study of the innovative development of a simulator for epidural anesthesia training in dogs

**DOI:** 10.3389/fvets.2024.1322871

**Published:** 2024-06-26

**Authors:** Paloma Lobo Moraes, Lianna Ghisi, Anna Júlia B. Paes de Barros, Vithor Hugo de Carvalho Peixoto, Pedro Eduardo Brandini Népoli, Edson Moleta Colodel, Luiz Felipe Souza de Lima, Roberto Lopes de Souza

**Affiliations:** Small Animal Surgical Center, Veterinary Hospital, Federal University of Mato Grosso (HOVET/UFMT), Cuiabá, Brazil

**Keywords:** 3D printing, veterinary education, anesthesiology, simulation, practical skills, phantom, training

## Abstract

Epidural anesthesia in dogs is a locoregional anesthesia technique used in veterinary medicine, becoming an important integrated application in the anesthetic protocol to provide safer and more effective analgesia to patients. For this, professionals must adhere to rigorous guidelines and possess technical skills. In this context, in veterinary education, the development of practical clinical skills represents a crucial aspect in the training of these professionals. However, traditional teaching methods have proven insufficient to ensure a consistent level of competence among recent graduates. The introduction of non-animal alternatives for educational purposes has contributed to the development of simulation-based teaching, an innovative and accessible field capable of enhancing pre-clinical proficiency in students and reducing the use of live animals and cadavers. Despite its application in various areas of veterinary education, there are no conclusive results regarding the development of accessible simulators capable of effectively enhancing training in epidural anesthesia in dogs. Therefore, this article represents a pioneering study aimed at sharing a method for creating SimuVet, a realistic simulator for training epidural anesthesia in dogs. The simulator was fully developed by veterinary researchers with limited experience in 3D printing and, after preliminary analysis, demonstrated excellent performance and ultrasonographic anatomy. Future work will focus on the formal validation of this simulator with the aim of improving the teaching and learning process for students and experts in performing epidural anesthesia in companion animals.

## 1 Introduction

Epidural anesthesia in dogs is a technique that serves as an excellent resource for providing more effective analgesia to patients. In summary, the approach is preferably performed through the lumbosacral space (between the seventh lumbar vertebra and the first sacral vertebra), relying on tactile feedback to ensure the correct positioning of the needle. Ultrasonography can also be employed to enhance the success of the procedure, especially in obese patients. Despite its importance, this procedure is challenging and requires healthcare professionals to possess well-developed practical clinical skills, while exercising caution in adhering to rigorous guidelines to minimize the risk of complications (1–5).

Throughout history, the teaching of practical skills in small animals has often involved the use of live patients, cadavers, or the observation of clinical cases. However, due to ethical and economic concerns, efforts are being made to implement alternative methods in conducting studies, aiming to reduce the use of live animals, in order to minimize suffering and replace *in vivo* tests. Because of that, the teaching opportunities have been limited, as they depend on exposure to cases and require the consent of guardians. Since skill development is intrinsically linked to the amount of deliberate practice time, this directly impacts the level of proficiency students achieve. While training on live patients is the ideal scenario for learning the epidural anesthesia technique, the absence of pre-clinical skills exposes patients to the risk of injury. The conflicting situations experienced by students during training make it challenging to develop their skills, as stress and anxiety can hinder the learning process. In some cases, this may even discourage students from participating in teaching sessions. Therefore, the acquisition of these practical skills becomes a crucial factor in the training of competent professionals in the practice of epidural anesthesia in domestic animals (6–9).

The intersection of these pedagogical, ethical, and economic factors associated with the challenges of traditional teaching has led educational and research institutions to explore simulation technology and other non-animal methods for educational purposes. In the context of veterinary education, simulators are devices or simulated conditions created specifically to replicate real-life scenarios, allowing students to practice their skills without relying on live animals. Simulation-based teaching represents a relatively recent and innovative area of study that can significantly improve the learning process, reduce the use of live animals and cadavers, and thus promote animal welfare (6, 7, 10).

Although high-fidelity simulators are available in the market, their high acquisition and maintenance costs limit their widespread adoption in medical education overall. With technological advances achieved in recent decades, 3D printing has gained prominence and quickly become a versatile and affordable teaching tool. The combination of simulation technology with 3D printing allows the creation of realistic and cost-effective simulators, offering an innovative solution to help educators overcome limitations found in traditional teaching methods. Simulators can be developed and used in various ways to facilitate different types of learning. Among these approaches, practical simulators based on models and mannequins are considered the most valuable resource for teaching the practical skills required for this procedure. Despite the widespread application of simulation technology in various educational domains, there is still a shortage of studies related to its use in anesthesiology, both in the medical and veterinary fields (6, 7, 10–23).

As enthusiasts of 3D printing technology, we were motivated to develop SimuVet, a simulator capable of enhancing the technical proficiency of veterinary trainers at a minimal cost. The name ‘SimuVet’ is a term created in Portuguese, combining the words ‘simulation’ and ‘veterinary,’ reflecting our goal to provide an innovative, realistic, and affordable tool for training in epidural anesthesia in dogs.

## 2 Methods

This experimental study involved the collection of qualitative data during the creation and analysis of the ultrasound images of the simulator. The data were then presented in the form of categorical variables, which were summarized in tables. The research was exempt from submission to the Animal Use Ethics Committee since it did not involve the use of live vertebrate animals, in accordance with Law No. 11,794 of October 8, 2008.

In summary, SimuVet was developed using computed tomography (CT) data from a canine cadaver. The spine and mold were created through 3D printing, and various materials were used to simulate the skin, muscle, and ligamentum flavum, such as a transparent synthetic gelatin and sealing silicone. To initiate the validation process of SimuVet, practical tests and preliminary ultrasound analyses were conducted to support our hypothesis that the simulator can provide a realistic training experience.

All production and analysis steps were carried out at the Veterinary Medicine School of the Federal University of Mato Grosso (FAVET/UFMT) by the authors themselves. We selected a canine cadaver that was stored in a cold room, originating from the Animal Pathology department of the Veterinary Hospital (HOVET/UFMT). The cadaver was positioned in sternal recumbency, placed on a rigid foam support, with the pelvic limbs positioned cranially to simulated the recommended posture for epidural anesthesia, allowing for better identification of anatomical landmarks. It was destined for disposal, weighed 11.3 kg, had a good body condition score (3/5), and showed no deformities in the spine, making it suitable for the procedure.

The cadaver underwent a tomographic examination using the SOMATOM Spirit multislice CT scanner (Siemens Healthcare Headquarters, Erlangen, Germany) in the Imaging Diagnostics department. The examination followed the bone window capture protocol, with an adjustable field of view suitable for the region’s size, settings of 130 kV, 70 mAs, 2.0 mm slice thickness, 1.65 mm pitch, and helical capture technique (2, 5, 16). The tomographic acquisition followed the bone window capture protocol, with an adjustable field of view, settings at 130 kV, 70 mAs, 2.0 mm slice thickness, 1.65 mm pitch, and helical capture technique. After the procedure, the cadaver was returned to the respective department.

The exam data was saved in the Digital Imaging and Communication in Medicine (DICOM) format and later segmented using the inVesalius software (CTI, DF, Brazil) with semi-automatic segmentation technique. This process resulted in the creation of a 3D digital replica of the lumbosacral region. Subsequently, we used the Meshmixer software (Autodesk, San Rafael, CA, United States) to customize the bones that would be 3D printed from the lumbosacral region. Our goal at this stage was to enhance image resolution and, consequently, the accuracy of anatomical structures, minimizing the print area as much as possible without altering the actual size of the cadaver’s anatomy.

**Figure 1:**
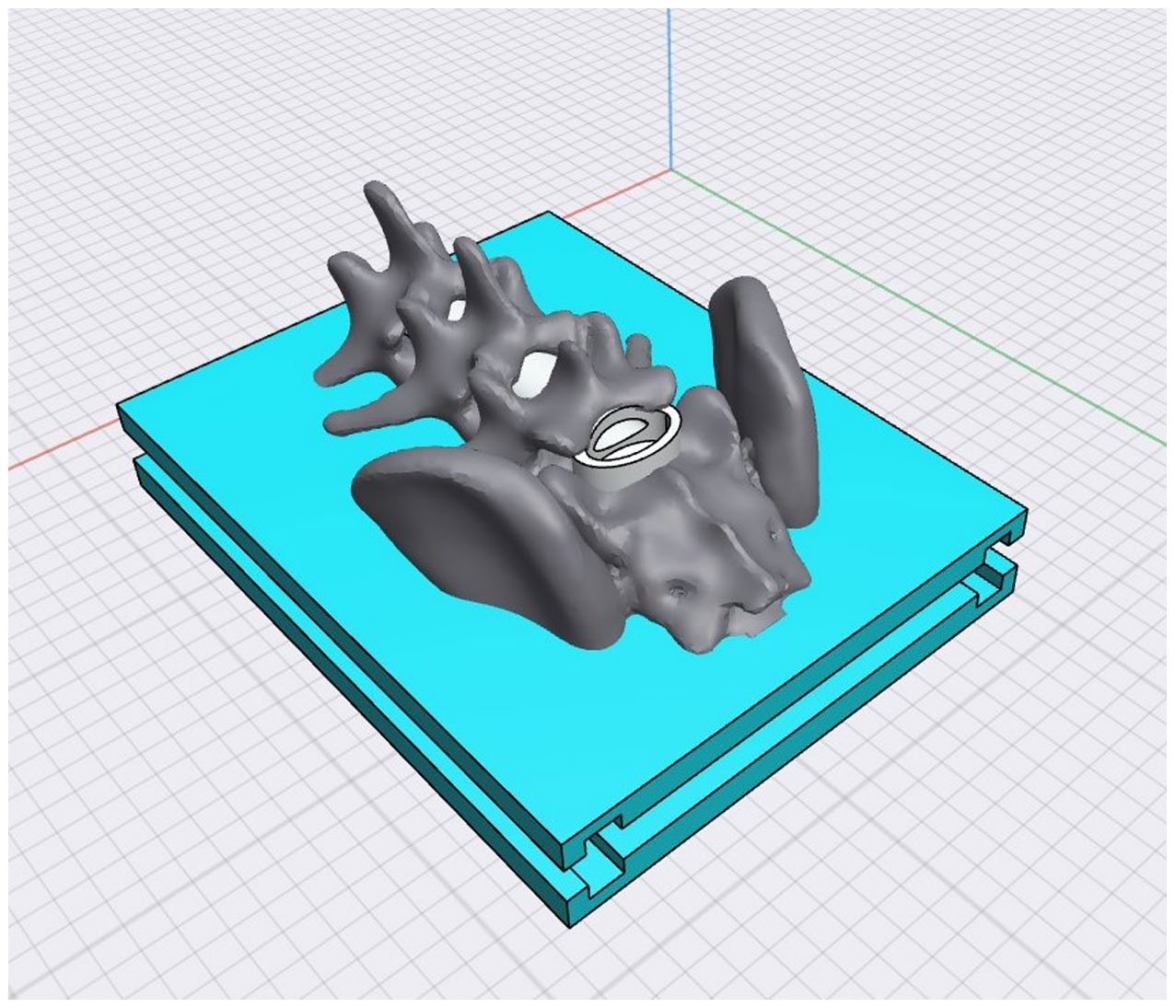
The SimuVet model created digitally in 3D using Shapr3D software. This is the model that will be 3D printed, consisting of the mold base represented by the blue area; the gray area represents the lumbosacral anatomical region obtained from CT; the white area inside the medullary canal represents the cannula that will accommodate the injected solution to simulate the volume of injected anesthetic. The 3D printed mold according to the previously created 3D digital model. The printing was done with white ABS filament; it is possible to observe the base, the lumbosacral spine, and the cannula. This image represents the SimuVet at the stage of the mold ready to receive the ballistic gelatin. It is possible to observe in the lumbosacral joint region a transparent membrane covering the lumbosacral space; this membrane is the sealing silicone applied manually to simulate the yellow ligament. The lateral parts were also 3D printed and are fitted into the SimuVet, receiving silicone application between the mold fittings to ensure sealing.

For the mold design process, we obtained a free educational license for Shapr3D software (Shapr3D Zrt, Budapest, Hungary). Within this software, we constructed a cannula within the medullary canal to ensure its integrity even after immersion in gelatin. This cannula was designed to accommodate the physiological solution that would be injected during practice with SimuVet. A single comprehensive 3D digital object was generated by combining the base of the mold, the 3D spine, and the cannula (see Figure 1A). The sides of the mold were designed with fittings to allow disassembly after the completion of the fabricated structures.

The Ultimaker Cura 5.1.1 software (Ultimaker, Utrecht, Netherlands) was used to specify the printing settings. The mold components were printed separately using a 3D printer with fused deposition modeling (FDM) GTMax3D Core A3 v2 (GTMAX3D Ltda., SP, Brazil), equipped with 1.75 mm thick acrylonitrile butadiene styrene (ABS) printing filament. The printing configuration included a print speed of 100 mm/s, 20% infill, a layer height of 0.15 mm, a nozzle temperature of 230°C, and a bed temperature of 110°C. After the completion of printing, support structures and excess filaments were removed (see Figure 1B).

Before constructing the SimuVet, various materials and techniques were tested and selected based on available literature to effectively simulate the primary tissues and anatomical structures involved in the procedure, including skin, muscles, fat, and ligaments (13, 15, 17, 18, 20). After several rounds of successive tests and trial and error, we established the method that will be outlined below.

**Figure 2:**
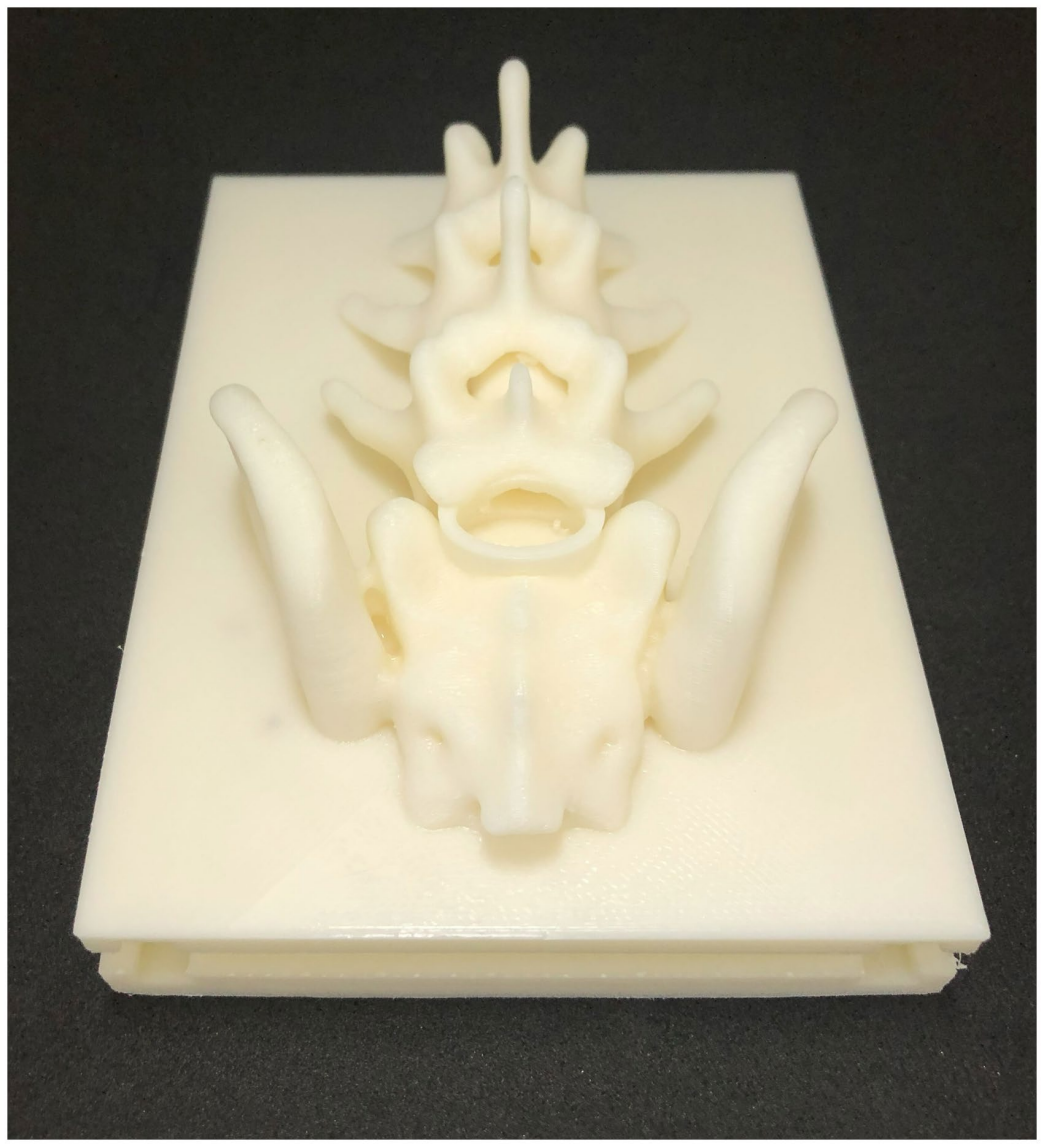
Completed SimuVet model. After the ballistic gelatin fully cooled, the removable parts of the mold were taken off, and the silicone synthetic skin layer was placed on top. The lower and rigid layer represents the SimuVet’s base, with visible openings where the lateral parts fit for gelatin remolding. Just above, a thicker layer represents the finalized ballistic gelatin simulating muscle tissue. The topmost, thinner layer on the SimuVet represents the silicone rubber layer simulating the skin.

To enhance realism and facilitate the identification of palpable anatomical landmarks, we used computed tomography (CT) images to determine the appropriate thickness for the epidermal and muscular tissues. We opted for a synthetic skin thickness of 3 mm and a ballistic gelatin thickness of 1 cm, starting from the iliac crests. To simulate the skin, we used a silicone rubber product from Redelease (Redecenter Materiais Plásticos Acessórios LTDA, São Paulo, SP, Brazil) and created a 3 mm thick layer large enough to cover the top of the model (see in Figure 2).

To simulate the yellow ligament, we used transparent sealing silicone from Pulvitec Ltda. (SP, Brazil). This material is commonly used in the construction industry to seal hydraulic connections. It is a low-cost material readily available in the market, known for its excellent elasticity and adhesion, short curing time, and easy handling. To create the ligamentum flavum, we applied it manually under the lumbosacral joint, which was a simple and quick process. Additionally, we applied silicone in the spaces between the removable parts of the mold to seal them. After a few hours of curing, all the applied silicone dried completely and remained adhered (see Figure 1C).

To simulate the muscular tissue, we used commercial synthetic ballistic gelatin from Lab37 (Guarulhos, SP, Brazil) and followed the fusion and remolding instructions provided by the supplier. Once the ballistic gelatin reached its melting point, the solution was poured gradually into the mold to minimize the formation of bubbles (15). The mold was then allowed to cool and solidify over several hours. Subsequently, the mold’s side parts were removed, and the layer of synthetic skin was positioned under the gelatin. This resulted in the completion of the SimuVet, ready for use (see Figure 2).

Following the design and manufacturing process, our team conducted a brief evaluation of the realism and tactile sensation of the model. We proceeded with practical tests guided by ultrasound. The ultrasound equipment used for this procedure was the Mylab Six Cristalline (Esaote SpA, Genoa, Italy). The region was examined using a micro-convex transducer with a frequency of 3 MHz, following the instructions of a previous study in which the authors performed the epidural anesthesia technique in live and cadaveric dogs under ultrasound guidance (4).

## 3 Results

Despite the challenges faced, which included a small team with limited experience in 3D printing technology, we successfully created a simulator for epidural anesthesia in dogs. This achievement became possible using DICOM data obtained from a single cadaver, along with an FDM 3D printer and readily available materials. Obtaining the DICOM images played a crucial role in our approach, allowing realistic simulation of the spine with precise topography of the lumbosacral joint in 3D while the cadaver was in sternal recumbency. Additionally, the tomographic examination guided the creation of synthetic skin and ballistic gelatin, resulting in a high-fidelity simulation of tissue thicknesses. We firmly believe that this method allowed an authentic reproduction of palpation of the spinous processes, highlighting the pedagogical potential of SimuVet as a high-fidelity training tool for epidural anesthesia procedures in dogs.

**Figure 3:**
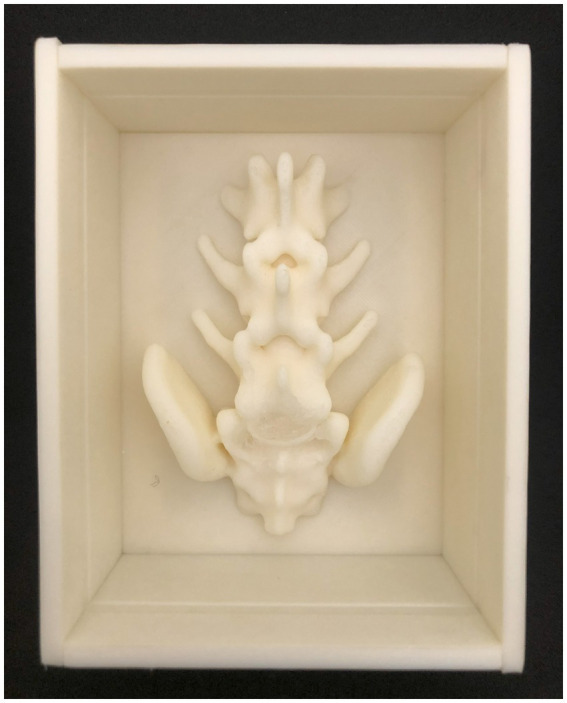
Ultrasound Images of SimuVet during the Tuohy Needle Passage. An ultrasound image of the model in a parasagittal section during the needle passage, as indicated by the dashed line and the yellow label ‘Needle Tuohy.’ Parallel hyperechoic lines can be observed; the lumbosacral space is indicated by the asterisk (*), where a hyperechoic circle represents the ligamentum flavum. Immediately cranially, a hyperechoic line represents the spinous processes of the seventh lumbar vertebra, and immediately after the lumbosacral space, the hyperechoic line represents the sacral vertebrae.

**Figure 4:**
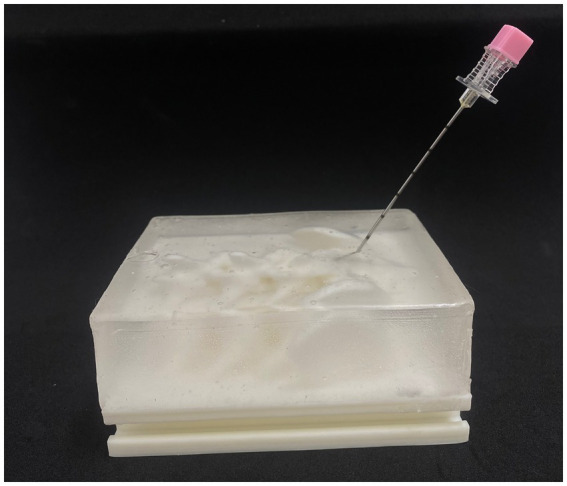
Image showing the needle insertion in the lumbosacral space without the skin lawyer for a better view of the SimuVet.

The 3D model printing was successfully completed in a total time of 24 h and 53 min, resulting in a total weight of 215 g of ABS plastic, as detailed in Table 1. The entire 3D printing and manufacturing process took approximately 2 days and resulted in a final cost of R$ 311.66. This cost includes expenses related to the materials used but does not include costs associated with the equipment already available at the institution. All details regarding costs and estimated production time can be found in Table 1. The ultrasound images showed reference points with very similar echogenicity to the lumbosacral ultrasonographic anatomy of dogs. The ability to visualize the spinous processes of L7, sacral vertebrae, and the ligamentum flavum with very similar echogenicity in a live animal, along with the ability to track the needle progression in real-time (1, 4), these are notable features that attest to the effectiveness of SimuVet in generating realistic ultrasound images, as shown in Figures 3, 4.

**Table 1:** Breakdown of costs and approximate manufacturing time for SimuVet.

Cost (R$)		Production time	
3D printing	24,72	3D printing	Base: 11 h 38 min Side parts: 13 h 14 min
Ballistic Gelatin	248,54	Ligamentum flavum	12 h 20 min
Silicone Rubber	3,40	Ballistic Gelatin	Fusion and Remolding: 1 h 30 min Cooling: 3 h
Sealing Silicone	35,00	Synthetic Skin	1 h 15 min
Total	311,66		42 h 57 min

In the preliminary tests conducted by the authors, during which the complete simulation of the Tuohy needle technique and injection of physiological solution to represent the anesthetic were performed, we observed excellent tactile feedback. This included the distinctive sensation of the needle passing through the ligament, known as the ‘POP’ sensation, and loss of resistance, in addition to providing an excellent anatomical representation during the ultrasound examination.

At this point, we have conducted a preliminary analysis of the feedback provided by veterinarians who volunteered to perform the technique with SimuVet. It is important to note that, although the data have not been published yet and are undergoing in-depth statistical analysis, the initial impressions strongly indicate that the simulator has substantial potential to enhance the teaching and learning process of the epidural anesthesia technique in dogs.

SimuVet was tested with 20 veterinarian participants. Among these participants, 10 were anesthetists, while the other 10 had little or no experience with locoregional anesthesia. All participants performed the simulation using the Tuohy needle and injected saline solution. Considering that some participants made more than one attempt during the simulation, SimuVet allowed at least 22 needle passes without any visible harmful changes. An interesting feature was that, at a certain point, we were able to recover the injected saline solution by inserting a catheter attached to a syringe into the side of the lumbosacral space without compromising the Tuohy needle insertion area. We proceeded with the tests of the other participants using the recovered solution.

Although these data have not been fully analyzed statistically and far less published, we can state that all evaluations were very positive. We received additional feedback from volunteer participants, highlighting that prior training on the phantom facilitated the execution of the technique on a cadaver due to the greater ease of palpation and visualization of anatomical landmarks during simulation. Only one volunteer, who was the last participant, rated the sensation of loss of resistance as “poor.” However, they provided a comment stating that despite not feeling the loss of resistance in the model, the technique provided satisfactory skills for execution on the cadaver during training.

Thus, it can be inferred that SimuVet not only has the potential to enhance the development of practical skills for veterinary students and experts but can also contribute significantly to more ethical learning by reducing the use of live animals and cadavers in education. Our next phase involves conducting a comprehensive statistical analysis of the results to validate our hypotheses.

## 4 Discussion

SimuVet represents a remarkable achievement, especially considering that it was conceived by veterinary researchers with limited experience in 3D printing. This accomplishment was possible thanks to technological advances, the reduction in 3D printing equipment costs, the availability of free software, and access to scanning and printing equipment in our institution. This approach not only substantially reduced production costs but also simplified material handling and significantly shortened production time, aligning with findings from existing literature (6, 10–15, 17–19, 21–33).

Certainly, high-fidelity simulation models are commercially available for medical education, covering applications in both human and veterinary fields. However, their adoption in educational and research institutions has been hindered by high acquisition and maintenance costs, as well as other reported drawbacks (7, 17, 34, 35). The rise of 3D printing technology has revolutionized the production of practical simulators, making them affordable, highly accessible, and with a lower environmental impact. This has stimulated the development of new studies and the integration of modern methodologies by institutions and educators worldwide, leading to the incorporation of simulators to overcome pedagogical limitations associated with conventional methods (6, 7, 11, 12, 16, 19, 21, 25, 29, 32, 36).

Despite these advances, research on the use of simulators in animal anesthesia is still relatively limited. One study, for instance, found that among 73 reviewed articles on the use of simulators for teaching practical skills to veterinary students, only 2 articles focused on simulation models for regional anesthesia. Furthermore, of these, only one article specifically addressed the teaching of epidural anesthesia in dogs (6, 23).

This research involved the development of a 3D-printed canine bone anatomical simulator and an investigation into its efficacy in training veterinary students in the epidural technique. The study yielded promising results but also faced notable limitations. Among the participants, 45% successfully executed the epidural technique. While there was no statistically significant difference in the success rate, the group that used the simulator reported feeling more confident and encountered less difficulty during the procedure. However, this same group expressed greater difficulty in identifying the ligamentum flavum during the procedure. Several challenges were identified that affected the effectiveness of epidural anesthesia training with the simulator. These included the absence of indicators confirming needle insertion into the epidural space, the lack of negative pressure feedback, and the absence of a sense of resistance on the plunger. These factors hindered the simulator’s ability to fully replicate the tactile feedback experienced during an actual epidural procedure (23). Epidural anesthesia is often practiced relying on tactile feedback, where the operator identifies the lumbosacral space through the palpation of anatomical landmarks and ensures correct needle placement for epidural injection (1–5). While replicating all anatomical layers in a simulator presents a significant challenge, incorporating these elements becomes essential to provide a more realistic training experience (13, 15, 17, 18, 20, 23, 34).

The selection of materials for our manufacturing process was informed by previous studies related to building simulators for human anesthesia. After several rounds of iterations and experiments, we chose to incorporate the materials that performed best in our assessment. The decision to use CT data was supported by an extensive body of literature highlighting the significance of 3D printing based on DICOM data from actual patients. This approach allows for the rapid creation of digital models with high resolution in a matter of minutes (13, 15, 17, 20, 27, 33, 37–39). Furthermore, the images generated from DICOM data played a crucial role in our approach, primarily because they contributed to the precise palpation of anatomical landmarks. The inclusion of removable skin and a transparent synthetic gelatin matrix was deliberate, as it facilitated the visualization of needle location and trajectory. When simulating the ligamentum flavum, the incorporation of sealing silicone proved effective for several reasons. These include its low cost, wide availability on the market, ability to generate realistic ultrasonographic images, and capacity to provide tactile feedback during needle insertion, with subtle contrast in resistance between the gelatin and the ligament. This combination of features strengthens the didactic potential of SimuVet, enabling realistic training in the epidural anesthesia technique.

One highlight is the efficiency of its manufacturing, taking approximately 25 h of printing and about 2 days of practical assembly. This efficiency compares favorably to similar human anesthesia simulators, with printing times ranging from 13 h to 3 days and practical assembly periods ranging from 1 to 5 days (13, 15, 17, 20, 23). It is important to note that the wide variation in costs among these anesthesia simulators reflects the time, infrastructure, and knowledge required for their production (16, 17, 22, 27, 28). The most expensive component of the simulator is the ballistic gelatin, accounting for 79.74% of the total cost. It is important to highlight that acquisition costs can vary significantly depending on the region and the time when this material is purchased; here in Brazil, we used 289 g of ballistic gelatin, totaling R$ 248.54. Comparatively, a study from 2019 conducted in the USA used 4.7 kg of synthetic gelatin but spent only USD $153 (15). Although it implies a more significant initial investment, ballistic gelatin offers good cost-effectiveness for SimuVet. Its strength, which withstands repeated needle passes, and its remolding and reuse capability significantly contribute to the simulator’s longevity.

SimuVet offers numerous advantages considering the environmental, ethical, and financial concerns associated with practical skills training that involves live animals and cadavers. We believe it has the potential to significantly reduce reliance on animals in the teaching-learning process, thereby promoting the development of more confident and skilled professionals. SimuVet should not be seen as a replacement for traditional teaching but as a valuable complementary tool that enhances the learning experience (6–8, 18, 23, 27, 34, 40). In summary, SimuVet represents an economical and ethical alternative for veterinary anesthesia training, considering both the time and resources required for its creation.

Our initial impressions of SimuVet have been overwhelmingly positive, underscoring its unique ability to deliver realistic training experiences. SimuVet incorporates essential features for an effective epidural simulator, including the representation of palpable structures, tactile feedback, visualization of needle trajectory, and excellent ultrasonographic anatomy. These factors lead us to believe that SimuVet stands as an exceptional educational tool. As far as our knowledge goes, the combination of these visual and tactile elements has not been achieved in any other study related to animal epidural anesthesia simulators (1, 4, 6, 23, 34). This makes our study an innovative contribution, as it shares the creation of an accessible and one-of-a-kind epidural simulator in veterinary medicine.

The core purpose of SimuVet is to promote ethical education without compromising the quality of student learning. This approach offers a viable alternative to enhance the development of practical skills among students and residents in a safe and cost-effective manner. We hope that our study can serve as an inspiration for other educators and researchers to consider the adoption of simulation-based teaching in veterinary education. Our future work will concentrate on validating SimuVet based on the results gathered from our research with veterinarians. We believe that these results will inform enhancements to its performance and motivate its implementation in Brazilian and international teaching and research programs, especially in institutions that may lack access to traditional simulation resources.

## Data availability statement

The original contributions presented in the study are included in the article/supplementary material, further inquiries can be directed to the corresponding authors.

## Ethics statement

The requirement of ethical approval was waived by Comissão de Ética no Uso de Animais da Universidade Federal de Mato Grosso for the studies involving animals because The research was exempt from submission to the Animal Use Ethics Committee since it did not involve the use of live vertebrate animals, in accordance with Law No. 11,794 of October 8, 2008. The studies were conducted in accordance with the local legislation and institutional requirements. Written informed consent was not obtained from the owners for the participation of their animals in this study because Only one canine cadaver that was destined for disposal, originating from the Veterinary Hospital of the Federal University of Mato Grosso, was selected for this research. As it is a teaching hospital, the owners sign an agreement allowing the study of the animals treated in the hospital, which is attached to their respective medical records. The cadaver underwent a tomographic examination and was then returned to the responsible department.

## Author contributions

PL: Writing – review & editing, Writing – original draft, Visualization, Project administration, Methodology, Investigation, Conceptualization. LL: Writing – review & editing, Resources, Methodology, Conceptualization. LG: Writing – review & editing, Supervision, Resources, Project administration, Methodology. PN: Writing – review & editing, Supervision, Resources, Methodology. EC: Writing – review & editing, Supervision, Resources, Methodology. RS: Writing – review & editing, Visualization, Supervision, Resources, Project administration, Methodology. VC: Writing – review & editing. AB: Writing – review & editing.
